# Turning the tide on Alzheimer’s disease: modulation of γ-secretase

**DOI:** 10.1186/s13578-021-00738-7

**Published:** 2022-01-04

**Authors:** Joanna E. Luo, Yue-Ming Li

**Affiliations:** 1 Chemical Biology Program, Memorial Sloan Kettering Cancer Center, New York, NY 10065 USA; 2 Program of Pharmacology, Weill Graduate School of Medical Sciences of Cornell University, New York, NY 10021 USA

**Keywords:** γ-secretase, Alzheimer’s disease, Inhibitor, Modulator, Mechanism

## Abstract

Alzheimer’s disease (AD) is the most common type of neurodegenerative disorder. Amyloid-beta (Aβ) plaques are integral to the “amyloid hypothesis,” which states that the accumulation of Aβ peptides triggers a cascade of pathological events leading to neurodegeneration and ultimately AD. While the FDA approved aducanumab, the first Aβ-targeted therapy, multiple safe and effective treatments will be needed to target the complex pathologies of AD. γ-Secretase is an intramembrane aspartyl protease that is critical for the generation of Aβ peptides. Activity and specificity of γ-secretase are regulated by both obligatory subunits and modulatory proteins. Due to its complex structure and function and early clinical failures with pan inhibitors, γ-secretase has been a challenging drug target for AD. γ-secretase modulators, however, have dramatically shifted the approach to targeting γ-secretase. Here we review γ-secretase and small molecule modulators, from the initial characterization of a subset of NSAIDs to the most recent clinical candidates. We also discuss the chemical biology of γ-secretase, in which small molecule probes enabled structural and functional insights into γ-secretase before the emergence of high-resolution structural studies. Finally, we discuss the recent crystal structures of γ-secretase, which have provided valuable perspectives on substrate recognition and molecular mechanisms of small molecules. We conclude that modulation of γ-secretase will be part of a new wave of AD therapeutics.

## Introduction

Alzheimer’s disease (AD) is the most common cause of dementia affecting more than 6 million Americans. In 2021, AD and other dementias cost $355 billion in healthcare, and these costs could exceed $1 trillion by 2050 [1]. Early symptoms include memory loss and behavioral changes; in late stages of AD cognitive decline interferes with most everyday activities. While acetylcholinesterase inhibitors and N-methyl-D-aspartic acid (NMDA) antagonists alleviate cognitive and behavior symptoms [2], there are no treatments which delay or stop disease progression. Earlier this year the FDA approved aducanumab, the first novel therapy for AD in almost two decades. Aducanumab, a human monoclonal antibody which targets aggregated amyloid-beta (Aβ), reduced amyloid plaques in the brain, and is expected to delay cognitive decline [2, 3].

AD pathology is characterized by the deposition of Aβ plaques in brain tissue [4]. While the underlying disease mechanisms are complex and still being elucidated, multiple lines of evidence support the “amyloid hypothesis,” which posits that the accumulation of Aβ peptides initiates a chain of pathological events, including formation of neurofibrillary tangles and inflammatory responses, leading to widespread neurodegeneration and ultimately AD [5, 6]. The gene encoding the amyloid precursor protein (APP) was identified on chromosome 21, which corresponded with Down’s syndrome individuals who consistently exhibited AD [7, 8]. Mutations in *APP*, *Presenilin-1* (PS1), and *Presenilin-2* (PS2) have been linked to early-onset familial AD (FAD), which begins before age 60–65 [9–11]. APP mutations clustered at or near sites of APP proteolytically processed by secretases to promote amyloidogenic Aβ [12–14]. *PS1* and *PS2* mutations were demonstrated to directly affect APP cleavage by γ-secretase and cause toxic gain of function to increase the ratio of Aβ42/Aβ40 [15, 16]. Finally, advances in brain imaging and cerebrospinal (CSF) biomarker studies on AD patients have shown that the presence of Aβ precedes about two decades or more before other pathological characteristics [17].

The amyloid hypothesis has spurred many treatment strategies which aim to reduce Aβ in the brain, but none had improved clinical outcomes. While aducanumab validates the approach for Aβ-targeted therapies, the complex, multi-faceted etiology of AD compels the need for better understanding of pathological mechanisms and identification of therapeutic targets for multiple safe and effective AD treatments. Therefore, in this review, we introduce γ-secretase as a compelling target and summarize the development and mechanistic studies of small molecules which target γ-secretase.

### γ-secretase: a relevant target

**Fig. 1:**
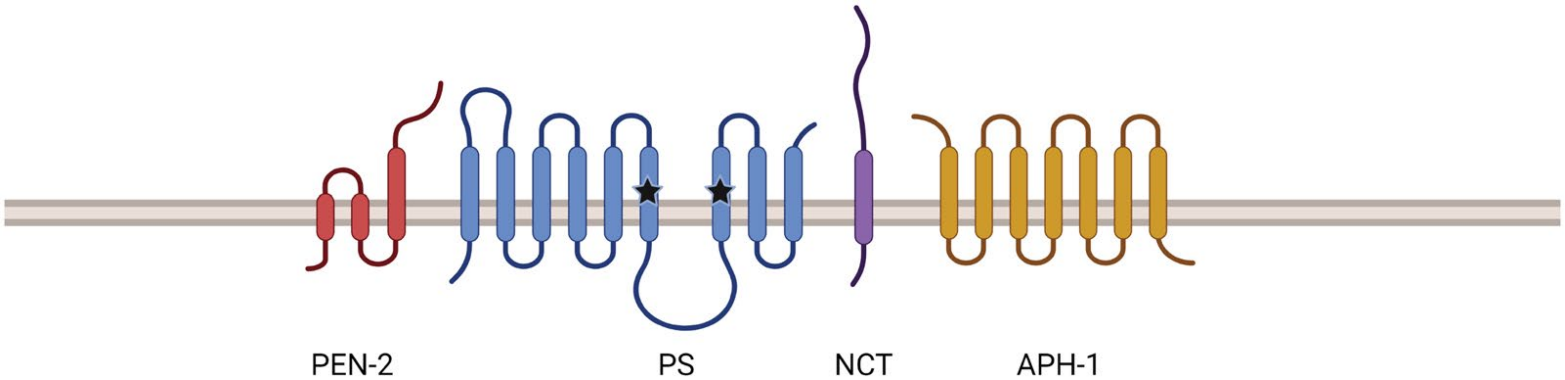
γ-secretase and its obligatory subunits. Catalytic aspartyl residues are indicated by stars. Adapted from “Gamma secretase” by BioRender.com (2021)

γ-secretase is an intramembrane aspartyl protease composed of four essential subunits: Presenilin (PS), Nicastrin (NCT), Anterior pharynx defective 1 (Aph-1), and Presenilin enhancer-2 (Pen-2) (Fig. 1) [18, 19]. PS, encoded by two isoforms *PS1* and *PS2,* is the catalytic subunit of the γ-secretase complex [20, 21]. The two transmembrane domain aspartates are required for endoproteolysis of PS into N-terminal (NTF) and C-terminal (CTF) fragments [22]. Both fragments remain associated as a stable heterodimer, and both are required for catalytic activity [20, 22]. Nicastrin and Aph-1 are responsible for substrate recognition, trafficking, and assembly of the γ-secretase complex. Pen-2 facilitates the proteolysis of full length PS into PS-NTF and PS-CTF to activate γ-secretase [19, 23].

**Fig. 2:**
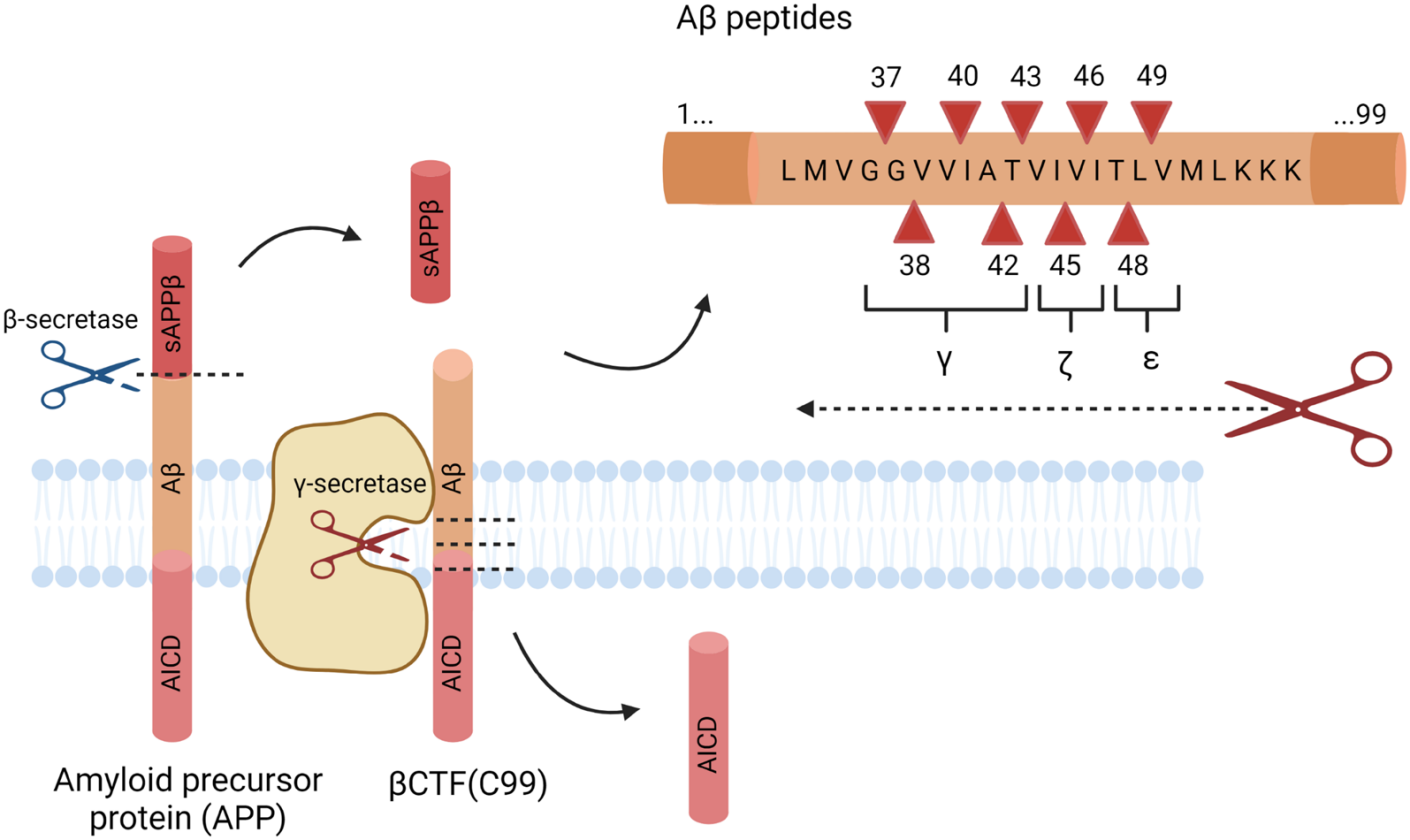
APP processing by γ-secretase. β-secretase cleaves APP to generate βCTF (C99) and release soluble APP (sAPPβ). Afterwards γ-secretase cleaves βCTF between Aβ49 or Aβ48 (ε-cleavage), resulting in two product lines. Further processing (ζ- and γ- cleavages) generates Aβ peptides released into the extracellular space. Created with BioRender.com

After cleavage by β-secretase produces the C-terminal fragment of APP (C99), γ-secretase cuts C99 at multiple sites to generate heterogeneous Aβ species (Fig. 2) [24]. Initial proteolysis (ε-cleavage) between amino acids 50 and 49 results in the successive trimming (ζ- and γ- cleavages) from Aβ49 46 43 40 37 peptides, while cleavage between amino acids 49 and 48 results in trimming from Aβ48 45 42 38 peptides [25, 26]. The isoforms Aβ40 and Aβ42 have been implicated in AD; of the two species, Aβ42 is more prone to aggregation and considered the more pathogenic species [27, 28].

Mutations in *PS1* and *PS2* have been linked to early-onset familial AD. To date more than 300 mutations for PS1 and PS2 have been reported [29, 30]. While the mechanism by which these mutations cause FAD pathogenesis has been widely investigated, two theories have been proposed: the amyloid hypothesis and the presenilin hypothesis. The amyloid hypothesis suggests that *PS1* and *PS2* FAD increase Aβ42 production, which leads to neuronal cell death and dementia. These mutations are observed to increase the ratio of Aβ42/Aβ40 [31]; the relative increase in Aβ42 production is more prone to aggregation and formation of amyloid fibrils in the brain [28]. The presenilin hypothesis suggests that loss of presenilin function in the brain triggers FAD. Presenilin was demonstrated as essential for learning, memory, and neuronal survival [32].

### Regulation of γ-secretase

γ-Secretase is regulated by many intricate mechanisms, ranging from the assembly of active and mature complexes to spatial compartmentalization and lipid composition [33, 34]. Because only a small fraction of γ-secretase complexes are catalytically active [35], it was hypothesized that other co-factors could stimulate the inactive pool of γ-secretase. Discoveries of γ-secretase modulatory proteins (GSMPs), non-essential subunits which can bind to and modulate γ-secretase in response to cellular and environmental changes, have added an interesting layer of regulation [33, 34]. Multiple studies have identified GSMPs which regulate γ-secretase activity and substrate specificity and are dependent on specific contexts: GSAP by aging [36, 37], IFITM3 by innate immunity and aging [38], Hif-1α by hypoxia [39], and SERP1 by ER stress [40]. GSMPs therefore have become implicated in the development of therapeutics for AD.

### Learning from γ-secretase Inhibitors

Targeting γ-secretase has been challenging due to its wide range of γ-secretase substrates. γ-secretase cleaves type I integral transmembrane proteins after shedding of their ectodomains. While over 149 putative substrates have been reported [41], APP and Notch are the most characterized. Notch signaling is crucial for cell fate decisions during development, the maintenance and differentiation of neuronal stem cells [42, 43]. After cleavage by furin-like protease in the Golgi and ADAM metalloproteases at S1 and S2 respectively, Notch is cleaved by γ-secretase at S3 (analogous to the ε-cleavage site of APP) to release the Notch intracellular domain, which translocates to the nucleus and acts as a transcription factor to activate target genes [44].

**Fig. 3:**
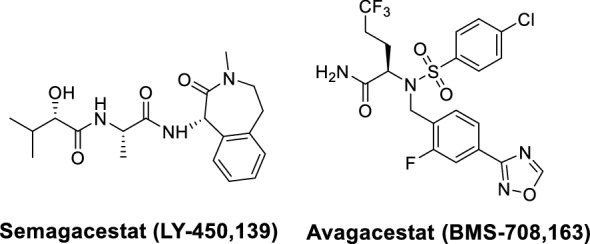
Structures of γ-secretase inhibitors in AD clinical trials

γ-Secretase inhibitors (GSIs) failed in clinical trials due to nonselective inhibition of substrates. Semagacestat and Avagacestat are among the most widely known cases (Fig. 3). Semagacestat (LY-450,139) terminated in phase III due to increased risk for skin cancer, associated with inhibition of Notch1 signaling, and cognitive worsening [45–48]. In dosing and kinetic studies, the high concentration of Semagacestat given during the once a day regimen likely resulted in “bursts” of full inactivation which lead to inhibition of Notch and other substrates [49].

Avagacestat (BMS-708,163) was referred to as “Notch-sparing” inhibitor. However, it was terminated in phase II due to increased risk for skin cancer and gastrointestinal distress [50, 51]. Mechanistically, the specificity of Avagacestat for APP and Notch has been questioned [52, 53]. While GSIs did not succeed in AD clinical trials, due to their inhibition of Notch signaling several GSIs have been pursued in clinical trials for various cancers [54–56]. Their applications as chemical probes have also been valuable for advancing our understanding of the structure and regulation of γ-secretase [57]. Recently, an imaging probe based on semagacestat demonstrated high specificity and increased uptake in tumor mouse models, suggesting that such tracers could be used to monitor γ-secretase inhibition and target engagement in clinical responses [58].

### The Finesse of γ-secretase modulators

**Fig. 4:**
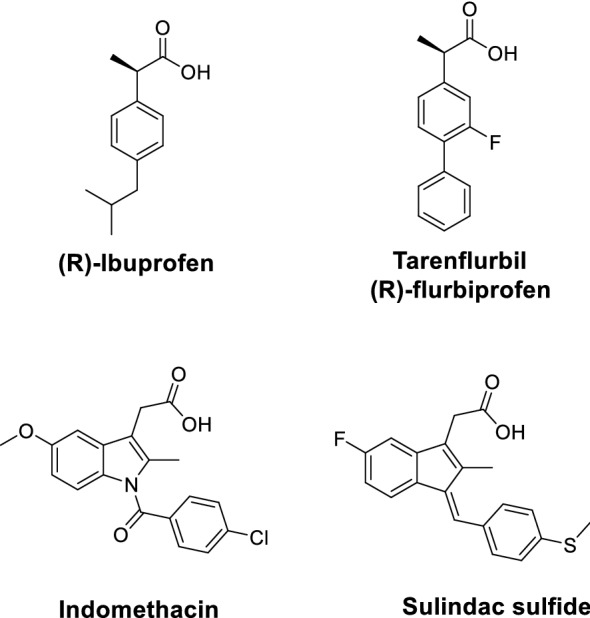
Structures of representative first generation γ-secretase modulators

The shifting approach from global inhibition t subtle modulation of γ-secretase has resulted in the development of γ-secretase modulators (GSMs). Weggen et al. first characterized a subset of NSAIDs, such as ibuprofen, indomethacin, and sulindac sulfide, which selectively reduce levels of the pathogenic Aβ42 in favor of the shorter and less pathogenic Aβ38 without inhibition of Notch (Fig. 4) [59]. These effects were separate from their inhibitory effects of cyclooxygenase (COX) activity and so were considered the first generation GSMs. However, these NSAIDs displayed weak in vitro potency and poor brain penetration and entered clinical trials with limited success [60]. Tarenflurbil (R-flurbiprofen), with Aβ42 IC_50_ ~ 200–300 μM, slowed cognitive decline in patients with mild AD in phase II, but did not achieve clinical outcome in phase III [61].

**Fig. 5:**
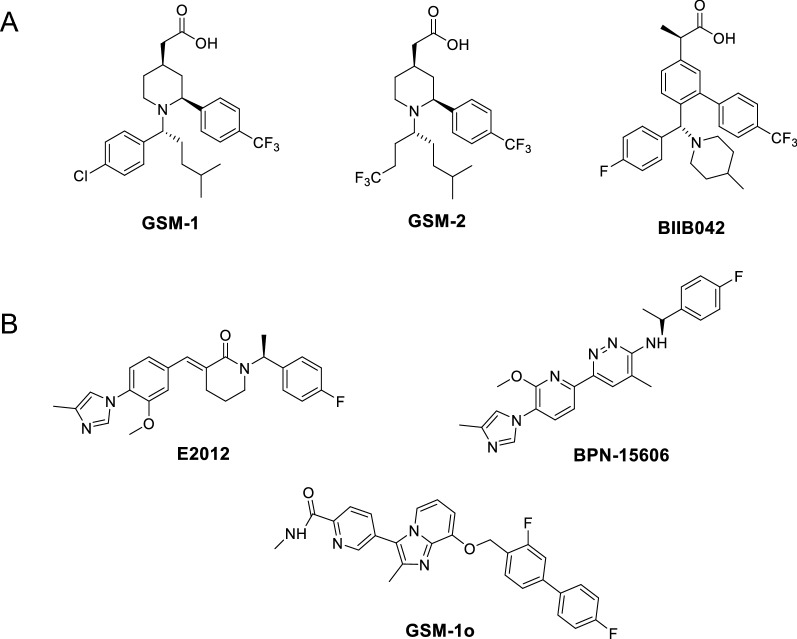
Structures of representative second generation γ-secretase modulators (A) Carboxylic acid NSAID-derived GSMs and (B) Heterocyclic non-NSAID derived GSMs

Second generation GSMs were developed to improve upon these parameters. They are divided into two categories: (1) carboxylic acid NSAID-derived GSMs and (2) heterocyclic non-NSAID derived **GSMs (Fig. ****5****). The** development of second generation GSMs has been extensively reviewed elsewhere [60, 62, 63]. In brief, carboxylic acid GSMs reduce levels of Aβ42 without affecting Aβ40 while simultaneously increasing Aβ38. They were developed through substitution of the core aryl ring with piperidine ring and optimization of the substituents on piperidine to generate a series of piperidine acetic acid GSMs (Fig. 5A).

Heterocyclic GSMs reduce levels of Aβ40 and Aβ42 while increasing Aβ37 and Aβ38. E2012 was the first non-NSAID GSM to enter clinical trials. It was temporarily halted due the observation of cataracts in a 13-week rat safety study, but after no ocular toxicity was seen in subsequent safety studies in rats and monkeys, the clinical trial was allowed to proceed [64]. E2012 reduced plasma levels of Aβ40 and Aβ42 in a dose-dependent manner in healthy patients [65], but was not developed further. The compound possesses a key arylimidazole moiety that has since served as the foundation for other imidazole-based GSMs (Fig. 5B) [62, 63]. Industry groups have also investigated scaffolds outside arylimidazole to improve drug-like properties [66–68]. The overall challenges in the development of small molecule GSMs have been improving potency and brain penetration while alleviating high lipophilicity, cytochrome P (CYP) inhibition, and human ether-a-go-go related genes (hERG) inhibition [62, 63]. Methods such as the central nervous system multi parameter optimization (CNS MPO) score [69] and ligand-lipophilicity efficiency (LLE) have been employed to improve the likelihood of identifying drug-like compounds.

**Fig. 6:**
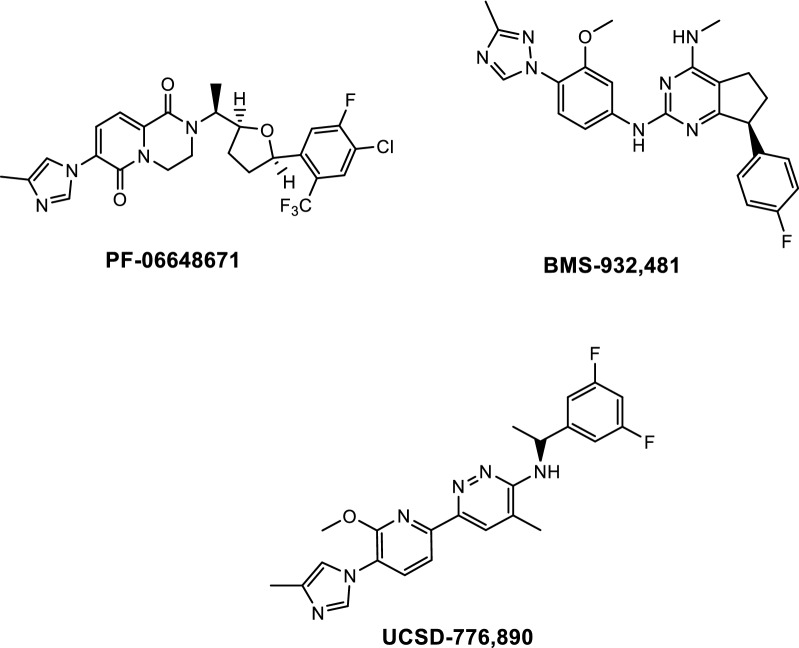
Structures of γ-secretase modulators in clinical trials

Several recent GSM candidates are described here (Fig. 6). A study from Pfizer investigated PF-06648671, derived from bicyclic pyridinones, in three phase I trials [70]. In 14 day single-dose and multiple-ascending daily doses in healthy normal subjects, the oral GSM was well tolerated. PF-06648671 dose-dependently lowered concentrations of CSF Aβ40 and Aβ42 and increased Aβ37 and Aβ38, with no change in total CSF Aβ. While these results support future dosing studies on PF-06648671, further clinical developments are currently unknown.

Scientists from Bristol-Myers Squibb reported the design and phase I studies for the bicyclic pyrimidine GSM BMS-932,481 [71, 72]. Single and long-term daily dosing studies demonstrated dose-dependent increases in CSF Aβ37 and Aβ38 and corresponding decreases in CSF Aβ40 and Aβ42 without changes in total Aβ. However, elevated alanine aminotransferase (ALT) levels, indicating liver toxicity, were also observed, and further development of BMS-932,481 was discontinued [72]. In the previous year, one of the oxidative products of BMS-932,481 was identified as the primary metabolite found in rat and human liver microsomes [73]. The authors hypothesized that the conversion of BMS-932,481 to this metabolite, in which an alcohol was substituted at the C-5 position, led to the formation of reactive species which could result in liver injury. Development of GSM derivatives from this group focused on blocking the metabolism at the C-5 position have yet to be reported.

A collaboration between the University of California San Diego and Massachusetts General Hospital synthesized and characterized a series of pyridazine-derived GSM analogs [74]. The top candidate UCSD-776,890 reduced Aβ40 and Aβ42 in a dose-dependent manner across acute, subchronic, and chronic dosing studies in multiple species. In prophylactic and disease-modifying regimens administered to 3 and 6 month AD transgenic mice respectively, UCSD-776,890 reduced plasma and brain Aβ40 and Aβ42 as well as amyloid deposition and microgliosis. Additionally, based on comparison of systemic exposure the compound at 50% effective equivalent human dose is expected to have over a 130-fold safety margin. These studies demonstrate the possibility for small molecule GSMs to be safely administered as secondary prevention in genetically predisposed subjects, or at-risk subjects who are amyloid-positive based on PET imaging. UCSD-776,890 is currently being prepared for phase I studies.

The ability to image amyloid and CSF biomarkers in human subjects has been crucial to monitoring the progress of clinical trials in AD [75]. A PET radiotracer based on pyridazine-derived GSM BPN-15606 demonstrated good brain uptake and selectivity for imaging PS1/ γ-secretase in brains of AD transgenic mice [76]. Elevated brain uptake in AD mice was observed in several critical regions, including the cortex, hippocampus, and mid-brain compared to wild-type mice. Interestingly, imaging studies in the brains of rodents and nonhuman primates revealed overlapping areas of higher uptake, pointing to conservation of γ-secretase activity. The GSM-based probe is a valuable molecular imaging tool which can be applied to further investigate physiological γ-secretase structure–function and potentially optimized as a radiotracer in AD patients.

### Chemical biology of γ-secretase

For many years, structural and functional insights of γ-secretase came from chemical probes derived from GSIs and GSMs [57, 77]. Photoaffinity labeling (PAL) has been a valuable tool for target identification of small molecules [78]. Photoaffinity probes, or photoprobes, contain a photoreactive group which crosslinks to binding targets upon UV irradiation and a reporter tag which enables purification or monitoring of the target. The alkyne handle has been the primary choice for reporter tag due to the ability to “click” on a biotin or fluorophore group using Cu-catalyzed azide-alkyne cycloaddition [57, 60].

The earliest photoprobes were based on transition state inhibitors directed at the active site of γ-secretase, such as L-685,458 (L458) and III-31-C [79, 80]. L458-based probes, which individually labeled subsites of the active site, identified PS1 as the catalytic subunit of γ-secretase [20]. A III-31-C-based probe was used in competitive labeling studies to characterize GSIs into different mechanistic classes [81]. More recently, the binding site of BMS-708,163 was mapped by photoprobes with cleavable linkers [82]. Peptide mapping using LC–MS/MS demonstrated that the BMS-708,163 probe inserted into L282 of PS1, which was confirmed with molecular dynamic simulations. L282 is located on the inhibitory loop near the endoproteolytic site required for ɣ-secretase activation, suggesting that BMS-708,163 acts as a pan inhibitor of ɣ-secretase. The report was consistent with previous studies that had challenged Notch-sparing mechanism of BMS-708,193 [52, 53].

As GSMs were developed, PAL was employed to identify their binding targets. GSM probes were incubated in HeLa membranes, and then UV irradiated to crosslink them to nearby protein targets and followed by click chemistry with biotin-azide. Biotinylated proteins were then captured with streptavidin beads and analyzed by Western blot. GSM-1 and GSM-2-based probes were found to label PS1-NTF in both reconstituted PS1 and native forms of the ɣ-secretase complex in HeLa membranes [83]. Their labeling was blocked by excess of the parent compounds, demonstrating the specificity of the probe for PS1. Furthermore, GSM-1 enhanced the labeling of the L458-based probe GY4-P1, suggesting that carboxylic acid GSMs modulate γ-secretase by allosterically binding to PS1 and altering the conformation of the active site.

**Fig. 7:**
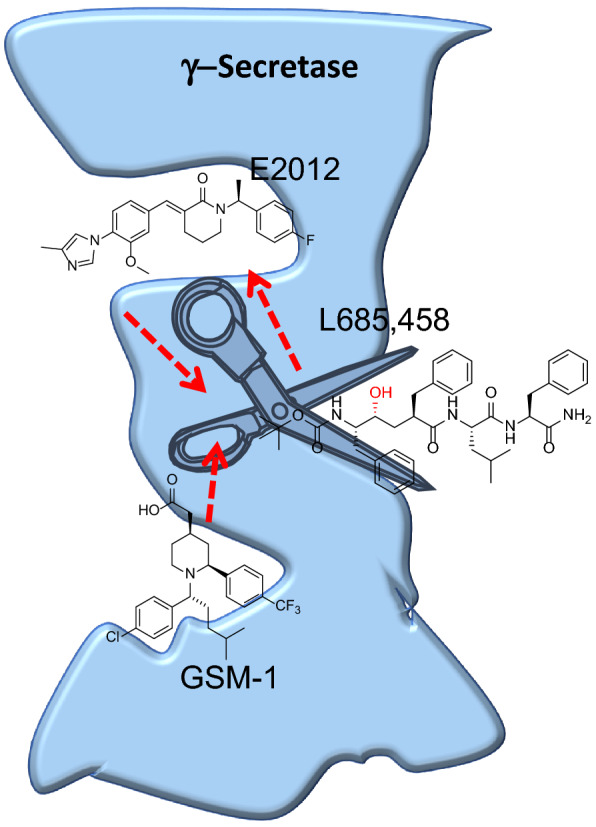
Multiple binding sites on PS1 established by photoaffinity labeling studies. L458 binds to the active site. GSMs such as GSM-1 and E2012 bind to allosteric sites and alter the conformation of the active site

Imidazole GSM-based probes RO-57-BpB and E2012-BPyne also labeled PS1-NTF in membranes and live cells [84, 85]. Competitive labeling by these probes revealed that GSMs and GSIs bind to multiple, distinct binding sites on PS1-NTF (Fig. 7). Furthermore, labeling of E2012-BPyne, but not acid GSM probes, was significantly enhanced in the presence of L458, which suggests the binding of L458 induces a more favorable conformation for E2012 to PS1. Together, the PAL studies on small molecule GSIs and GSMs have greatly improved our understanding of their mechanisms and laid the foundation for the subsequent molecule-bound crystal structures.

### Cryo-EM images of γ-secretase: a wellspring for drug design

Advances in cryo-EM have enabled detailed reports of the γ-secretase complex, with clear assignment of the transmembrane domains and precise location of the active site [86–88]. Structures of γ-secretase bound to APP and Notch have revealed key features of substrate recognition. Upon moving into the active site, the α-helix of the substrate transmembrane domain unwinds and extends into a β-strand to prepare for proteolytic cleavage. Many FAD mutations of PS1 line the substrate-binding cavity and while their mechanisms are unclear, they could alter substrate binding or unwinding. Finally, comparison of the two bound structures site showed notable differences in recognition by APP and Notch, which could be used as a framework to design substrate-selective inhibitors.

**Fig. 8:**
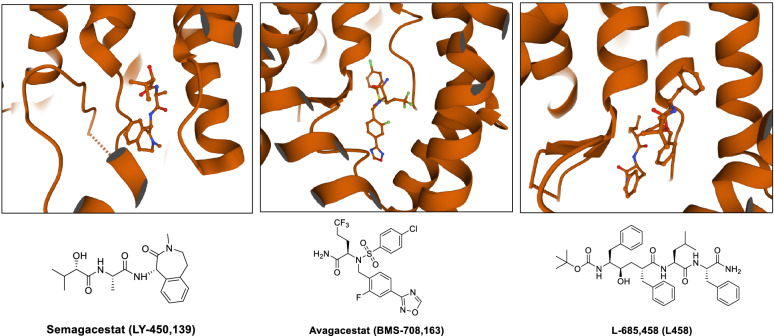
Visualization of γ-secretase bound to Semagacestat, Avagacestat, and L-685,458. PS1 is represented in orange. Protein Data Bank entries 6LR4, 6LQG, and 7C9I, respectively

Recently, the structures of γ-secretase bound to Semagacestat, Avagacestat L458, and the GSM E2012 have been reported [89]. The identification of their binding sites has helped elucidate the recognition and molecular mechanisms of these small molecules. Semagacestat, Avagacestat, and L458 occupy the same binding pocket in PS1 (Fig. 8) and overlap with the β-strand of APP and Notch. Their location suggests that the inhibitors block substrate recruitment into catalytic site. Displacing the substrate beta-strand could be a key strategy to designing more substrate selective GSIs. Key differences were also observed in recognition of the structurally distinct inhibitors. Comparing Semagacestat and Avagacestat, the binding of the bulkier Avagacestat induced more conformational changes to PS1 than Semagacestat binding. Additionally, L458 directly coordinated with the catalytic aspartate residues in PS1, confirming its role as a transition state inhibitor.

**Fig. 9:**
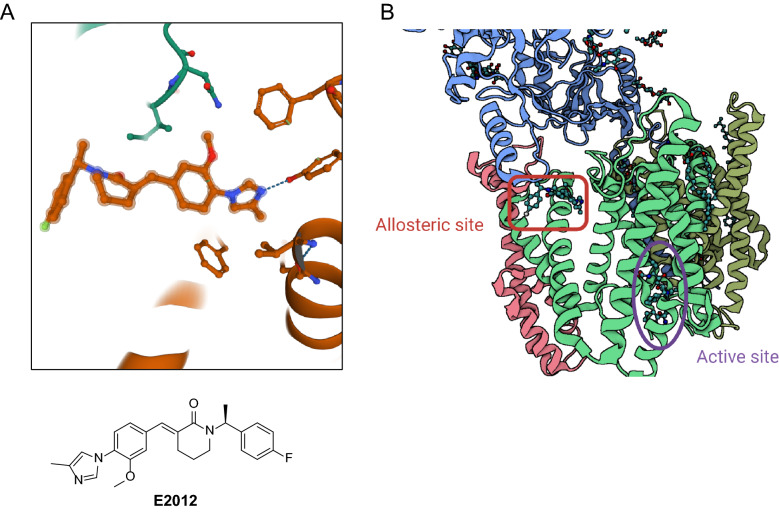
**A** Visualization of γ-secretase bound to E2012. PS1 is represented in orange and NCT is represented in green. Hydrogen bond between methylimidazole on E2012 and Tyr106 on PS1 is indicated by the dotted blue line. **B** Visualization of allosteric site and active site on γ-secretase. γ-secretase subunits represented are PS1 (green), Nicastrin (blue), PEN-2 (pink), and APH-1 (brown). Protein Data Bank entry 7D8X

E2012 was previously known to bind to an allosteric site on PS1 and enhanced binding of L458 [85]. Recognition of E2012 demonstrated the methylimidazole and phenyl groups inserted into a hydrophobic pocket between PS1 and NCT. E2012 was stabilized by a hydrogen bond between the methylimidazole and Tyr106 on loop-1 of PS1 (Fig. 9A). Loop-1 is known to interact with substrate proteins and coordinate between the substrate docking site and catalytic site, suggesting how GSMs can influence the active site of γ-secretase. Concurrent mutagenesis studies revealed that loop-1 is essential for γ-secretase’s processive cleavage and a critical binding site by heterocyclic GSMs [90].

Superimposing the E2012-bound γ-secretase structure in complex with an APP fragment revealed that the flurophenyl and piperidine groups clashed with APP transmembrane domain. Modifying any of the heterocycles on E2012 could improve binding affinity and/or selectivity for imidazole-like GSMs. GSMs bind to multiple allosteric sites on γ-secretase, which in turn may alter conformation of the active site (Fig. 9B) [85]. While these structural studies will need to be supported by experimental data, they can be applied towards rational design of the next generation GSIs and GSMs for AD therapeutics.

## Conclusion and future perspectives

While targeting γ-secretase has proven challenging, it should not diminish its potential as a crucial target for AD pathogenesis. The serious toxicities that halted clinical studies of GSIs demonstrated there were many knowledge gaps about γ-secretase biology and underestimation of its nuanced proteolysis before the compounds were evaluated in humans [49]. GSMs aim to stimulate γ-secretase’s carboxypeptidase-like trimming of Aβ peptides to their shorter, less pathogenic forms. Over the past two decades, industry and academic groups have optimized the potency and CNS penetration of GSMs, several of which have started clinical trials. The success of small molecule GSMs, as with other Aβ-targeted therapies, also depend on being administered in the early stages of AD pathology well before clinical manifestations. Amyloid-based biomarkers and diagnostics will be vital to identifying and monitoring trial subjects. The development of the first GSM-based radiotracer suggests that that γ-secretase expression could be monitored in AD patients.

In the past several years, detailed structures of γ-secretase have emerged. These structures have already been used in computational docking studies for GSMs [91]. Modeling GSMs with varying chemotypes could be insightful for comparing their mechanisms of recognizing and altering substrate and enzyme transmembrane domains. The latest γ-secretase structures bound to GSIs and E2012 offer a wealth of information for the design and lead optimization of more potent and substrate-selective small molecules. Drug combinations using drugs which act on distinct targets or show different mechanisms of action have been commonly been used in cancer [92]. A few combination therapies could involve (1) distinct structural classes of GSMs (2) GSMs and inhibitors of tau in neurofibrillary tangles, and (3) GSMs and agents which stimulate Aβ clearance.

Our understandings of γ-secretase regulation, structure, and function have built upon each other like a tide. The approval of the first Aβ-targeted therapy will undoubtedly bring in a new wave of AD therapeutics, and GSMs will be at the forefront.

## Data Availability

Not applicable.

## Abbreviations

AD: Alzheimer’s disease
Aβ: Amyloid-beta
APP: Amyloid precursor protein
PS1: Presenilin-1
PS2: Presenilin-2
FAD: Familial Alzheimer’s disease
CSF: Cerebrospinal fluid
NCT: Nicastrin
Aph-1: Anterior pharynx defective 1
Pen-2: Presenilin enhancer-2
NTF: N-terminal fragment
CTF: C-terminal fragment
C99: C-terminal fragment of APP
GSMP: γ-Secretase modulatory protein
GSI: γ-Secretase inhibitor
GSM: γ-Secretase modulator
COX: Cyclooxygenase
CYP: Cytochrome P
hERG: Human ether-a-go-go
CNS MPO: Central nervous system multi parameter optimization
LLE: Ligand-lipophilicity efficiency
ALT: Alanine aminotransferase
PAL: Photoaffinity labeling
